# Longitudinal Radiographic Outcomes and Revision Burden After Magnetically Controlled Growing Rod Treatment for Early-Onset Scoliosis

**DOI:** 10.3390/jcm15208049

**Published:** 2026-10-16

**Authors:** Yasar Unsal, Kadir Cetinkaya, Recai Kulak, Erva Eser, Ercan Bal

**Affiliations:** 1 Department of Neurosurgery, Ankara Bilkent City Hospital, Ankara 06800, Türkiye; 2 Department of Neurosurgery, Hitit University Çorum Erol Olçok Education and Research Hospital, Corum 19000, Türkiye; 3 Department of Biostatistics, Kırıkkale University, Kırıkkale 71450, Türkiye; 4 Department of Neurosurgery, Faculty of Medicine, Ankara Yıldırım Beyazıt University, Ankara 06800, Türkiye

**Keywords:** early-onset scoliosis, magnetically controlled growing rods, MAGEC, growth-friendly surgery, revision surgery, longitudinal outcomes

## Abstract

**Background:** To evaluate longitudinal radiographic outcomes, exploratory radiographic response, and planned versus unplanned revision burden after magnetically controlled growing rod treatment in patients with early-onset scoliosis. **Methods:** We retrospectively reviewed 55 patients with early-onset scoliosis treated with magnetically controlled growing rods at a single center. Complete year-3 radiographic measurements were available for 40 patients; therefore, year-3 estimates were conditional on imaging availability. Cobb angle, T1–S1 length, and apical vertebral translation (AVT) were assessed preoperatively and at postoperative years 1, 2, and 3. Longitudinal changes were evaluated using generalized estimating equations. Exploratory radiographic responder endpoints included ≥20° Cobb correction, ≥30% Cobb correction, and a ≥30 mm preoperative-to-year-3 T1–S1 length difference. Freedom from unplanned revision and composite endpoints were also evaluated. The radiographic thresholds were pragmatic and were not validated measures of clinically important improvement. **Results:** The cohort included 33 female and 22 male patients, with a mean age at surgery of 8.10 ± 1.86 years. Neuromuscular and congenital scoliosis accounted for 41.8% and 40.0% of cases, respectively, and dual-rod constructs were used in 83.6%. At year 3, mean Cobb angle was 40.15° ± 19.03°, mean radiographic T1–S1 length was 383.02 ± 54.42 mm, and mean AVT was 32.80 ± 23.56 mm among 40 patients with complete measurements. GEE models showed significant reductions in Cobb angle and AVT and increases in T1–S1 length at each postoperative year (all *p* < 0.001). At year 3, ≥20° Cobb correction, ≥30% Cobb correction, and ≥30 mm preoperative-to-year-3 T1–S1 length difference were achieved in 50.0%, 55.0%, and 67.5% of patients, respectively; 35.0% achieved both radiographic thresholds and 25.0% achieved both thresholds with no recorded unplanned revision. Patient-level fields recorded any complication in 20.0%, unplanned revision in 16.4%, internal revision classification in 43.6%, and definitive fusion in 16.4%; these fields were not treated as mutually exclusive. **Conclusions:** MCGR treatment was associated with longitudinal radiographic improvement among patients with available measurements. The preoperative-to-year-3 T1–S1 difference includes the effect of index surgery and should not be interpreted as biological spinal growth alone. These findings suggest that MCGR success should be assessed not only by curve correction, but also by measured spinal-length gain and revision burden.

## 1. Introduction

Early-onset scoliosis (EOS) comprises a heterogeneous spectrum of spinal deformities diagnosed before 10 years of age and may adversely affect thoracic growth, pulmonary development, and global spinal alignment when progressive curves remain untreated [1]. The objective of growth-friendly surgery is to control coronal deformity and preserve spinal and thoracic growth until definitive fusion [1,2]. Traditional growing rods are a standard distraction-based strategy; however, repeated open lengthenings are associated with cumulative anesthetic exposure, morbidity, implant complications, and substantial treatment burden [2,3].

Magnetically controlled growing rods (MCGRs) reduce this burden by enabling noninvasive outpatient distraction while maintaining growth-friendly deformity control [3,4]. Early clinical series and comparative studies have shown that MCGR treatment can achieve meaningful coronal curve correction and spinal height gain in selected patients with EOS, while reducing the need for scheduled open lengthening procedures compared with traditional growing rods [3,4,5]. These advantages have established MCGR as an important surgical option in progressive EOS.

Nevertheless, the clinical effectiveness of MCGR treatment cannot be judged solely by the magnitude of initial radiographic correction. Durability of correction, maintenance of spinal height gain, and cumulative revision burden are equally important determinants of treatment success. Implant-related complications, including distraction failure, rod fracture, fixation failure, anchor pull-out, infection, and loss of correction, remain clinically relevant limitations [6,7,8]. Unplanned reoperations due to failed distraction, proximal foundation failure, rod breakage, or infection may offset part of the intended benefit of avoiding repeated planned open lengthenings [7]. Systematic review data have similarly demonstrated substantial complication and unplanned revision rates after MCGR treatment, supporting the need to evaluate outcomes beyond early coronal correction alone [8].

Longitudinal assessment is particularly important because MCGR construct performance may evolve over time. Progressive reduction in achieved distraction, often discussed as “diminishing returns,” may limit spinal height gain and contribute to treatment failure during longer follow-up [9]. In addition, scoliosis etiology, baseline curve magnitude, rod configuration, and previous growth-friendly surgery may influence both radiographic response and mechanical complication risk [6,8,10]. Therefore, studies integrating longitudinal radiographic outcomes with patient-level response measures and planned versus unplanned revision patterns are needed to better define the real-world effectiveness of MCGR treatment in EOS.

The present study aimed to describe longitudinal radiographic outcomes and revision burden after MCGR treatment for EOS in a single-center cohort. We evaluated serial Cobb angle, T1–S1 length, and apical vertebral translation measurements, together with year-3 radiographic changes and exploratory responder endpoints. Complications, revision procedures, and definitive fusion were summarized using separately recorded patient-level variables. We also described outcomes by scoliosis etiology and year-3 radiographic availability and explored the association between baseline Cobb angle and time to unplanned revision.

## 2. Materials and Methods

### 2.1. Study Design and Ethical Approval

This single-center retrospective cohort study evaluated longitudinal radiographic outcomes, exploratory radiographic response, and revision burden after magnetically controlled growing rod (MCGR/MAGEC) (MAGnetic Expansion Control; NuVasive, Audubon, PA, USA) treatment for early-onset scoliosis (EOS). Patients who underwent MCGR/MAGEC instrumentation between January 2020 and December 2024 were screened for eligibility. The Institutional Ethics Committee of Ankara Bilkent City Hospital approved this study (TABED 1-26-2713; 17 June 2026). Informed consent was waived due to the retrospective design.

### 2.2. Patient Population

Patients with EOS who underwent MCGR/MAGEC treatment at our institution and had available clinical and/or radiographic follow-up data were included. EOS was defined as scoliosis diagnosed before 10 years of age. A total of 55 patients constituted the final cohort.

Demographic, etiological, surgical, and clinical variables were retrospectively collected from medical records, operative reports, outpatient notes, and radiographic archives. Variables included age at index MCGR surgery, sex, scoliosis etiology, surgery date, follow-up duration, primary versus revision/conversion MCGR implantation, previous external revision or conversion history, rod configuration, number of rods, and previous growth-friendly surgery when available. Etiology was categorized as neuromuscular, congenital, idiopathic, or other/unclear, and rod configuration as single-rod or dual-rod instrumentation. Nutritional status variables, including BMI-for-age z-score and weight-for-age z-score, were not systematically available in the source records and were not analyzed.

The decision to perform MCGR implantation, select rod configuration and anchor strategy, and schedule subsequent procedures was based on patient characteristics, curve pattern, remaining growth potential, previous surgical history, and surgeon judgment. Postoperative distractions were performed during outpatient follow-up according to institutional practice and individualized decision-making. Because detailed distraction-session data were not consistently available, the analysis focused on serial radiographic outcomes and revision burden rather than achieved distraction per session. The institutional lengthening protocol consisted of outpatient distraction according to the treating surgeon’s schedule and individualized clinical/radiographic assessment. Detailed session-level distraction dates, achieved millimeters per session, and standardized frequency were not consistently available. Planned rod or system exchange was defined as a procedure recorded as part of the expected growth-friendly pathway; the specific operative indication for each exchange was not consistently documented and was therefore not inferred retrospectively.

### 2.3. Inclusion and Exclusion Criteria

**Figure 1:**
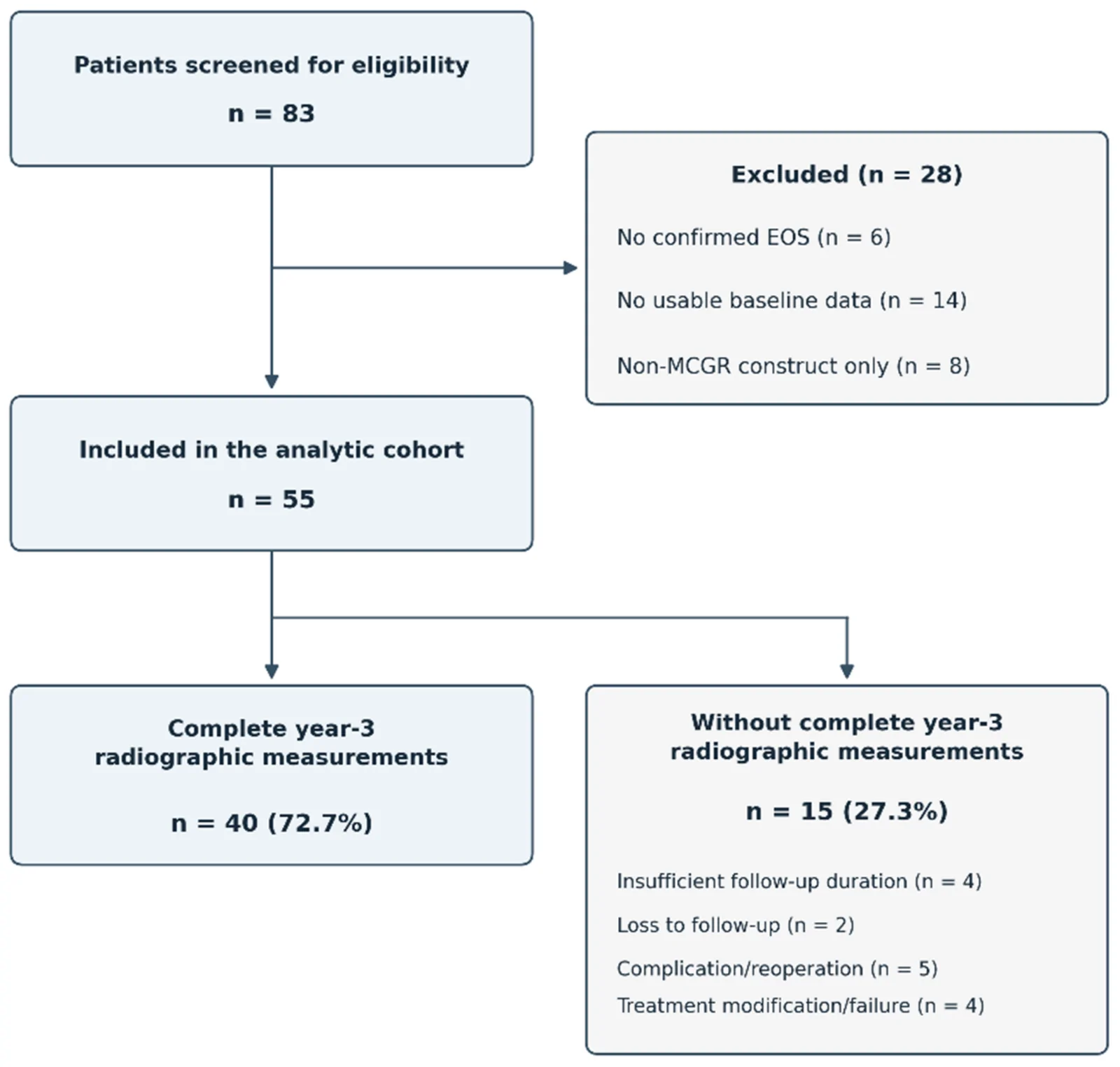
Patient selection and study cohort flowchart.

Patients were eligible if they had (1) confirmed EOS, (2) MCGR/MAGEC treatment at our institution, (3) available surgical and/or follow-up records, and (4) at least one evaluable preoperative radiographic parameter, including Cobb angle, T1–S1 length, or apical vertebral translation (AVT). The institutional screening log identified 83 patients who were assessed for eligibility for the present study. Of these, 28 were excluded before formation of the analytic cohort: six because EOS could not be confirmed, 14 because usable baseline data were unavailable, and eight because a non-MCGR construct was used exclusively. The final analytic cohort therefore comprised 55 patients (Figure 1).

### 2.4. Radiographic Assessment

Radiographic assessment was performed using routinely obtained standing whole-spine scoliosis radiographs and institutional radiographic records. The main parameters were Cobb angle, T1–S1 length, and AVT, recorded preoperatively and at postoperative years 1, 2, and 3 when available. Patients with partially available repeated measurements were retained in longitudinal analyses. Complete year-3 radiographic measurements were available for 40/55 patients (72.7%). Baseline characteristics, follow-up duration, and recorded outcomes were descriptively compared between patients with and without year-3 radiographs. The primary recorded reasons for unavailable year-3 radiographs were identified during the updated review of follow-up records and summarized as mutually exclusive categories. These reasons could not be reliably cross-tabulated by scoliosis etiology in the available extraction. Comparisons between patients with and without year-3 radiographs were descriptive; no inferential testing was performed. The comparison was interpreted cautiously because the unavailable group was small and the age field showed inconsistencies with date-derived age.

Cobb angle quantified coronal angular deformity, T1–S1 length served a radiographic measure of spinal height, and AVT assessed coronal translational alignment. All measurements were performed using Surgimap software (version 2.3.2.1) by a spine surgeon experienced in spinal deformity assessment.

### 2.5. Outcomes

The primary outcomes were longitudinal changes in Cobb angle and T1–S1 length from baseline to postoperative years 1, 2, and 3. Secondary outcomes included longitudinal changes in AVT, absolute and percentage Cobb correction at year 3, preoperative-to-year-3 and first-postoperative-to-year-3 T1–S1 length differences, and preoperative-to-year-3 AVT improvement. Exploratory radiographic responder endpoints were defined as ≥20° absolute Cobb correction, ≥30% Cobb correction, and a ≥30 mm preoperative-to-year-3 T1–S1 length difference. These pragmatic thresholds were used to describe the proportions of patients achieving specified radiographic changes and should not be interpreted as validated minimal clinically important differences. A dual radiographic response was defined as achievement of both ≥30% Cobb correction and a ≥30 mm preoperative-to-year-3 T1–S1 length difference. An additional composite endpoint combined this dual radiographic response with the absence of a recorded unplanned revision during follow-up. All year-3 responder analyses were restricted to patients with complete year-3 radiographic measurements.

### 2.6. Revision and Complication Assessment

Revision status was evaluated at two levels. Previous external revision or conversion history was recorded for patients who had undergone prior scoliosis or growth-friendly surgery elsewhere or underwent MCGR implantation as a revision/conversion procedure; these were considered baseline surgical-history variables and were not counted as post-index revision outcomes. All additional surgical procedures performed at our institution after index MCGR implantation were recorded as post-index internal revisions and classified as planned or unplanned.

Planned post-index revisions were procedures considered part of the expected growth-friendly treatment pathway, including planned rod exchange, system exchange, or final definitive surgery. Definitive fusion was considered an expected endpoint of growth-friendly treatment rather than a complication-related treatment failure. Because rod exchange, system exchange, definitive fusion, internal revision classification, external revision classification, and unplanned revision were recorded in separate fields that could overlap, they were not combined into a single planned-versus-unplanned procedure total. Unplanned revisions were procedures performed for unexpected clinical or radiographic problems, including infection, wound complications, implant failure, screw pull-out, rod fracture, system removal, loss of correction, or other unforeseen complications. Complications were recorded from medical records, operative notes, and follow-up documentation. Planned and unplanned revisions were reported separately to distinguish expected staged management from unexpected revision-related treatment failure.

### 2.7. Statistical Analysis

Continuous variables were summarized as mean ± standard deviation (SD) and, where appropriate, median and interquartile range (IQR). Categorical variables were summarized as counts and percentages. The number of available observations was reported for each outcome and time point because radiographic data availability varied during follow-up. Age recorded in months was converted to years by dividing by 12. Normality of within-patient differences was assessed using the Shapiro–Wilk test. Paired changes in Cobb angle, T1–S1 length, and apical vertebral translation (AVT) from preoperative measurements were evaluated at each postoperative assessment using the Wilcoxon signed-rank test. *p* values were adjusted using the Holm method across the four postoperative comparisons within each outcome. Longitudinal changes in Cobb angle, T1–S1 length, and AVT were assessed using separate generalized estimating equation (GEE) models. Time was entered as a categorical predictor, with the preoperative assessment as the reference category. Models used a Gaussian distribution, an identity link, an exchangeable working correlation structure, and robust sandwich standard errors. All available repeated measurements were included. Estimates are reported as mean differences with 95% confidence intervals (CIs). Because measurements were not available for every patient at every time point, the estimates may be affected if missingness was informative. Year-3 radiographic outcomes were calculated for patients with available year-3 measurements. Responder endpoints were summarized as counts and percentages; the thresholds were exploratory. Outcomes stratified by scoliosis etiology and by year-3 radiograph availability were summarized descriptively, without inferential subgroup comparisons. Unplanned revision-free survival was estimated using the Kaplan–Meier method and compared between baseline Cobb-angle groups (<60° and ≥60°) using the log-rank test. The ≥60° cutoff was exploratory. Separate univariable Cox proportional hazards models were used to estimate the hazard ratio (HR) for Cobb angle ≥ 60° versus <60° and the HR per 10° increase in baseline Cobb angle. Cox estimates were considered exploratory because few unplanned revision events were recorded. All tests were two-sided, and *p* < 0.05 was considered statistically significant. Holm-adjusted *p* values were used for the paired comparisons as described above. Statistical analyses were performed using R version 4.3.3 (R Foundation for Statistical Computing, Vienna, Austria). Figures were generated using Python version 3.12.14 and Matplotlib version 3.10.8.

## 3. Results

### 3.1. Patient Cohort and Follow-Up Availability

The cohort included 55 patients. Mean age at surgery was 8.10 ± 1.86 years; 33 patients (60.0%) were female and 22 (40.0%) were male. Neuromuscular scoliosis was present in 23 patients (41.8%), congenital scoliosis in 22 (40.0%), idiopathic scoliosis in 7 (12.7%), and other/unclear etiologies in 3 (5.5%). Dual-rod constructs were used in 46 patients (83.6%) and single-rod constructs in 9 (16.4%) (Table 1). Complete year-3 radiographic measurements were available for 40/55 patients (72.7%). The year-3 summaries and all derived year-3 response endpoints were therefore conditional on complete year-3 imaging.

Patient flow was as follows: 83 patients were initially identified; 28 were excluded before analysis (no confirmed EOS, n = 6; no usable baseline data, n = 14; non-MCGR construct, n = 8); and 55 patients were included in the final cohort. Among the 55 included patients, 40 (72.7%) had complete year-3 radiographic measurements and 15 (27.3%) did not. The primary recorded reasons for unavailable year-3 imaging were insufficient follow-up duration (n = 4), loss to follow-up (n = 2), complication/reoperation (n = 5), and treatment modification/failure (n = 4). Each patient was assigned to one primary reason category. Insufficient follow-up duration was distinguished from loss to follow-up, and absence of year-3 imaging was not itself considered evidence of treatment failure.

### 3.2. Longitudinal Radiographic Outcomes

**Figure 2:**
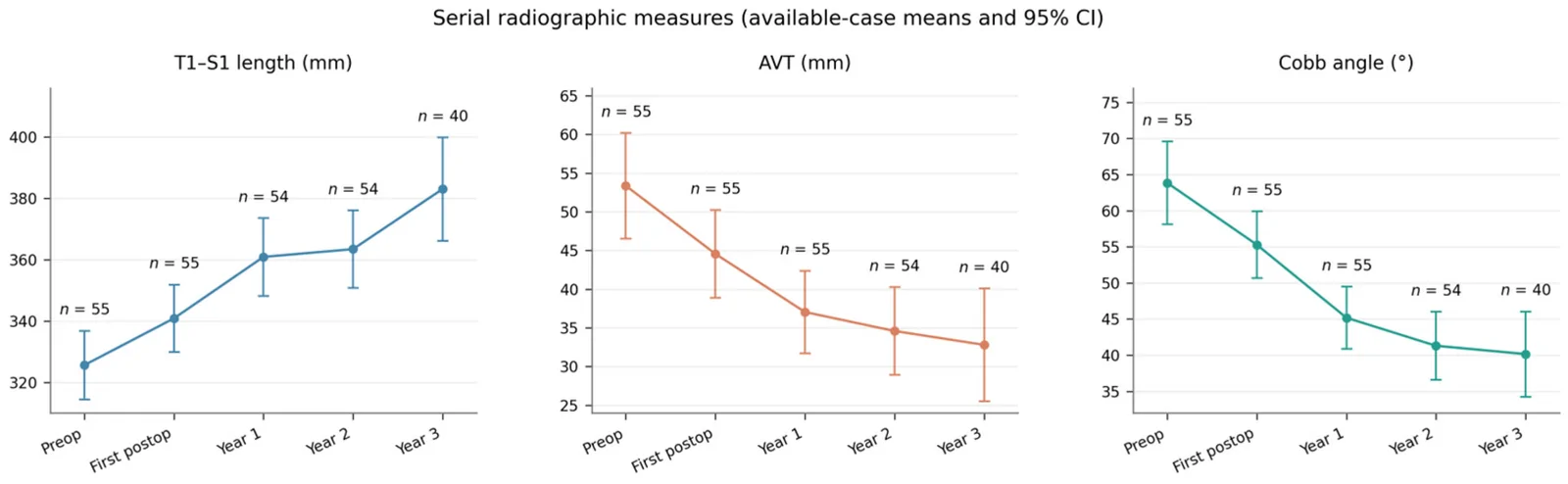
Serial radiographic measurements from the preoperative assessment to postoperative year 3. Points represent available-case means, and error bars indicate 95% confidence intervals for these means. The number of patients contributing to each estimate is shown at each time point. These descriptive summaries correspond to Table 2 and do not represent within-patient paired changes or GEE-estimated changes. Lower Cobb angle and apical vertebral translation (AVT) indicate improvement, whereas higher T1–S1 length indicates greater measured spinal length.

Radiographic measurements were available for all 55 patients at baseline and at the first postoperative assessment. At year 1, Cobb angle and AVT were available for 55 patients and T1–S1 length for 54; at year 2, 54 patients had Cobb angle, T1–S1 length, and AVT data; and at year 3, 40 patients had complete measurements for all three outcomes (Table 2, Figure 2).

Available-case mean T1–S1 length increased from 325.60 mm preoperatively to 340.84 mm at the first postoperative assessment and reached 383.02 mm at year 3. These descriptive means were calculated from the patients with measurements available at each time point; differences between these means should therefore not be interpreted as within-patient length changes. Cobb angle and AVT decreased, with most of the observed mean reduction occurring by year 1 and smaller decreases thereafter. These are available-case descriptive patterns; the year-3 summaries are conditional on measurements being available.

### 3.3. Paired Changes and GEE Models

Within-patient paired analyses demonstrated significant changes from preoperative values at the first postoperative assessment and at years 1, 2, and 3 for all three radiographic outcomes (all Holm-adjusted *p* < 0.001; Supplementary Table S1). At year 3, the median paired change was −20.00° for Cobb angle, +58.00 mm for T1–S1 length, and −19.50 mm for AVT among 40 paired observations. Longitudinal changes were evaluated using separate GEE models including all available repeated measurements from the 55 patients. Time was entered as a categorical predictor, the preoperative measurement was the reference, an exchangeable working correlation structure was used, and robust sandwich standard errors were calculated. Compared with preoperative values, Cobb angle and AVT decreased and T1–S1 length increased at each postoperative year (all *p* < 0.001; Table 3). Estimated year-3 changes were −23.70° for Cobb angle, +53.06 mm for T1–S1 length, and −21.11 mm for AVT.

### 3.4. Year-3-Derived Outcomes and Exploratory Response Endpoints

Among the 40 patients with complete year-3 radiographic measurements, mean Cobb correction was 22.93° ± 20.70°, mean percentage Cobb correction was 33.60% ± 28.33%, mean preoperative-to-year-3 T1–S1 length difference was 53.75 ± 40.01 mm, mean first-postoperative-to-year-3 T1–S1 length difference was 36.53 ± 37.73 mm, and mean AVT improvement was 20.33 ± 22.36 mm (Table 4). The preoperative-to-year-3 T1–S1 difference includes the index surgical change and subsequent measured change; it should not be interpreted as biological spinal growth alone.

The exploratory thresholds were achieved by 20/40 patients (50.0%) for ≥20° Cobb correction, 22/40 (55.0%) for ≥30% Cobb correction, and 27/40 (67.5%) for ≥30 mm preoperative-to-year-3 T1–S1 length difference. Both radiographic thresholds were achieved by 14/40 patients (35.0%), and both thresholds plus no recorded unplanned revision were achieved by 10/40 (25.0%).

### 3.5. Complications, Revision Fields, and Definitive Fusion

Eleven patients (20.0%) had a recorded complication. Infection-related complications were most frequent (8/11; 14.5% of the cohort), followed by screw pull-out (2/55; 3.6%) and rod fracture (1/55; 1.8%). Complication management included debridement in 6/11 patients, antibiotics in 2/11, screw revision in 2/11, and rod exchange in 1/11. Nine patients (16.4%) were flagged in the patient-level unplanned-revision field. Recorded indications were superficial infection/wound complications in 6/9, screw pull-out in 2/9, and rod fracture in 1/9. Internal revision classification was recorded for 24 patients (43.6%), external revision classification for 7 (12.7%), and no internal/external revision classification for 24 (43.6%). Rod exchange was recorded in 16 patients (29.1%), system exchange in 6 (10.9%), and definitive fusion in 9 (16.4%) (Table 5). These fields were recorded separately and may overlap; they were therefore not combined into a single planned-versus-unplanned procedure total.

### 3.6. Exploratory Outcomes by Etiology

Year-3 radiographs were available for 18/23 neuromuscular patients (78.3%), 16/22 congenital patients (72.7%), 6/7 idiopathic patients (85.7%), and 0/3 patients with other/unclear etiology. Any complication was recorded in 4/23 neuromuscular patients (17.4%), 4/22 congenital patients (18.2%), 3/7 idiopathic patients (42.9%), and 0/3 other/unclear patients. Unplanned revision was recorded in 3/23 neuromuscular patients (13.0%), 3/22 congenital patients (13.6%), 3/7 idiopathic patients (42.9%), and 0/3 other/unclear patients (Table 6). These subgroup summaries were descriptive; no inferential comparisons were performed because of small group sizes.

### 3.7. Comparison by Year-3 Radiographic Availability

Patients with year-3 radiographs (n = 40) and those without year-3 radiographs (n = 15) had broadly comparable baseline radiographic means. Mean age was 8.30 ± 1.86 versus 7.56 ± 1.81 years, mean preoperative Cobb angle was 63.08° ± 22.67° versus 65.93° ± 19.69°, mean preoperative T1–S1 length was 329.28 ± 43.17 versus 315.80 ± 40.01 mm, and mean preoperative AVT was 53.13 ± 26.79 versus 54.00 ± 23.99 mm. Recorded complication proportions were identical (20.0% in each group). The year-3-available group had longer recorded follow-up (45.38 ± 13.11 vs. 27.07 ± 4.03 months) (Table 7).

### 3.8. Exploratory Unplanned Revision-Free Survival Analysis

In the analysis, nine patients were classified as having an unplanned revision. Estimated unplanned revision-free survival for the overall cohort was 89.1%, 87.2%, and 82.6% at 1, 2, and 3 years, respectively. At 3 years, survival was 95.7% in the preoperative Cobb < 60° group and 70.8% in the Cobb ≥ 60° group; the log-rank *p* value was 0.017. In separate exploratory univariable Cox models, preoperative Cobb ≥ 60° was associated with a higher hazard of unplanned revision than Cobb < 60° (HR 8.30, 95% CI 1.04–66.44; *p* = 0.046). For each 10° increase in preoperative Cobb angle, the estimated HR was 1.28 (95% CI 0.96–1.69; *p* = 0.090) (Table 8). These estimates are hypothesis-generating because few events occurred and confidence intervals were wide.

## 4. Discussion

In this single-center longitudinal cohort of 55 patients with early-onset scoliosis treated with magnetically controlled growing rods, MCGR treatment was associated with longitudinal radiographic improvement among patients with available 3-year measurements. The principal findings were a mean year-3 Cobb angle of 40.15°, a mean radiographic T1–S1 length of 383.02 mm, and a mean AVT of 32.80 mm among the 40 patients with complete year-3 measurements. Model-based longitudinal analyses confirmed significant improvements in Cobb angle, T1–S1 length, and AVT at each postoperative time point compared with baseline. These findings support MCGR as a growth-friendly technique capable of providing coronal deformity correction, radiographic spinal-length increase, and improved coronal alignment in a clinically heterogeneous EOS population. However, these radiographic findings should be interpreted together with the cumulative surgical burden and incomplete follow-up. Patient-level records showed internal revision classification in 43.6%, unplanned revision in 16.4%, and definitive fusion in 16.4%; these fields were recorded separately and were not mutually exclusive. Definitive fusion represents an expected endpoint of growth-friendly treatment and should not automatically be interpreted as treatment failure. The cohort derivation identified 83 patients initially and excluded 28 before the final 55-patient analytic cohort. Complete year-3 radiographic measurements were available for 40 patients, whereas 15 did not have complete year-3 imaging. Baseline radiographic means and recorded complication proportions were broadly similar between patients with and without year-3 imaging, although the unavailable group had shorter recorded follow-up. This descriptive comparison reduces, but does not eliminate, concern about attrition or survivorship bias. Because no inferential comparison was performed and the unavailable group was small, year-3 findings and all responder rates remain conditional on continued availability of adequate radiographic follow-up.

Our radiographic improvements are consistent with previous MCGR studies [3,4,5,11,12,13,14,15]. In the present dataset, the mean preoperative-to-year-3 Cobb correction was 22.93°, the mean percentage Cobb correction was 33.60%, and the mean preoperative-to-year-3 T1–S1 length difference was 53.75 mm, paralleling prior reports of coronal deformity reduction and spinal height gain [5,10,11,12,13,14,15]. The observed T1–S1 length difference is clinically relevant because the aim of growth-friendly surgery is not only angular correction, but also preservation of longitudinal spinal and thoracic growth until skeletal maturity or definitive fusion becomes appropriate [1,2,12]. Nevertheless, the measured preoperative-to-year-3 difference includes the index surgical correction and should not be interpreted as biological spinal growth alone.

A key strength is the longitudinal evaluation of radiographic outcomes, as MCGR construct performance may change over time. Gardner et al. questioned whether the “law of diminishing returns,” originally described in traditional growing rods, also applies to MCGR lengthening [9]. Longer-term MCGR studies have emphasized that radiographic gains and implant-related events should be assessed across the entire treatment course rather than at a single endpoint [16]. Sankar et al. demonstrated progressively smaller T1–S1 gains with repeated lengthenings of dual growing rods [17]. In our cohort, the largest improvements in Cobb angle and AVT occurred early, whereas measured T1–S1 length continued to increase through year 3. The preoperative-to-year-3 T1–S1 length difference was 53.75 mm, whereas the first-postoperative-to-year-3 difference was 36.53 mm. This distinction is important: the former includes the index surgical correction, while neither measure can be interpreted as biological spinal growth alone without a complete distraction and imaging history. Accordingly, the present study reports measured radiographic length differences and T1–S1 length gain rather than pure biological growth.

Responder analyses were exploratory. Fifty percent achieved ≥20° Cobb correction, 55.0% achieved ≥30% correction, and 67.5% achieved a ≥30 mm preoperative-to-year-3 T1–S1 length difference. Both radiographic thresholds were achieved by 35.0%, and both thresholds plus no recorded unplanned revision by 25.0%. These proportions are descriptive and should not be interpreted as validated clinical success rates. The ≥20° Cobb, ≥30% Cobb, and ≥30 mm T1–S1 thresholds were pragmatic exploratory thresholds rather than validated minimal clinically important differences. Similarly, the 60° preoperative Cobb cutoff was used as a pragmatic descriptive stratification threshold, not as a validated or outcome-optimized cutoff.

Revision burden remains central when interpreting MCGR efficacy. The theoretical advantage of MCGR over traditional growing rods is reduction in scheduled open lengthening procedures [3,4,12]. However, MCGR constructs remain vulnerable to mechanical and biological complications, including rod fracture, distraction failure, anchor or foundation failure, infection, implant wear, and loss of correction [6,7,8,18,19,20,21,22,23,24]. In the present cohort, 83.6% of patients were free of a recorded unplanned revision, whereas 16.4% had an unplanned-revision flag. However, post-index internal revision classification was recorded in 43.6%, indicating that MCGR treatment remains surgically demanding even when unexpected reoperations are less frequent. This should not be interpreted as construct failure alone, because the revision fields were separate and most recorded procedure-type fields reflected the staged nature of growth-friendly treatment. In addition, 12.7% of patients had previous external revision or conversion history, representing baseline surgical complexity rather than a post-index outcome. Therefore, previous/conversion surgery, planned post-index revision, definitive fusion, and unplanned post-index revision should be reported separately to distinguish baseline complexity, expected treatment-pathway procedures, expected definitive treatment, and unexpected revision-related clinical events.

Our 20.0% complication rate was lower than several published series [6,8,10,13,19,20,21,25], possibly reflecting variability in definitions, techniques, and case mix. Moreover, biological and implant-related concerns such as wear debris, metallosis, and serum metal ion changes have emerged as important long-term issues in MCGR treatment [22,23,24]. These outcomes were not available in the present cohort but remain relevant for family counseling and long-term surveillance. The patient-level complication data showed 11 recorded complications: eight infection-related events, two screw pull-outs, and one rod fracture. Management included debridement, antibiotics, screw revision, and rod exchange. The timing of individual complications was not reliably preserved in the structured retrospective extraction and is therefore not interpreted in detail. Nine patients had an unplanned-revision flag, 24 had internal revision classification, and nine had definitive-fusion fields. These variables were not mutually exclusive and were recorded in separate data fields. Definitive fusion represents an expected endpoint of growth-friendly treatment and should not be equated with complication-related treatment failure.

The survival analysis suggested a possible association between greater baseline Cobb severity and unplanned revision, but this finding is exploratory and accompanied by wide confidence intervals. It should be considered hypothesis-generating rather than a validated clinical prediction rule Given the small number of unplanned-revision events, the Kaplan–Meier estimates and Cox associations should be interpreted cautiously, and the findings remain exploratory and hypothesis-generating. The baseline Cobb stratification should not be described as a clinical risk signal or prediction rule. Outcomes by scoliosis etiology were summarized descriptively. The small and heterogeneous etiological subgroups preclude firm conclusions regarding differences between etiologies. Year-3 radiographs were available in 18/23 neuromuscular patients, 16/22 congenital patients, 6/7 idiopathic patients, and 0/3 patients with other/unclear etiology. Any complication and unplanned revisions were more frequent numerically in the idiopathic subgroup, but these observations are descriptive and should not be interpreted as evidence of etiology-specific risk.

### Limitations

The study remains limited by its retrospective single-center design and incomplete year-3 imaging. Although the primary reasons for unavailable year-3 imaging were identified, their distribution across etiological subgroups could not be reliably established from the available extraction. Comparisons between patients with and without year-3 radiographs were descriptive, the unavailable group was small, and the age field contained inconsistencies. Some missing assessments were attributed to complications, reoperations, or treatment modification/failure, raising concern that imaging availability may have been related to the treatment course. Therefore, descriptive similarities in baseline characteristics do not exclude attrition or survivorship bias, and year-3 outcomes should be interpreted as conditional on radiographic availability. T1–S1 changes were measured radiographic length differences. Immediate surgical correction, subsequent distraction, and growth-related change could not be completely separated, and the preoperative-to-year-3 difference should not be interpreted as biological spinal growth alone. Responder cutoffs were pragmatic and exploratory rather than validated minimal clinically important differences. Only nine unplanned revisions were recorded, limiting the stability of survival and Cox estimates despite the use of robust descriptive interpretation. Separate revision, procedure-type, unplanned-revision, and definitive-fusion fields could overlap and could not be collapsed into a mutually exclusive procedure pathway. Patient-reported outcomes, pulmonary function, health-related quality of life, distraction compliance, implant wear, metallosis, and serum metal ion levels were unavailable [22,23,24]. Measurements were performed by a single surgeon using Surgimap, and interobserver reliability was not assessed. Baseline and longitudinal nutritional status, including BMI-for-age and weight-for-age z-scores, was not available. Therefore, the association between nutritional status and complications, revision surgery, follow-up completion, or change across etiological subgroups could not be assessed. The median follow-up by year-3 availability, exact timing of individual complications, and patient-level indications for planned exchanges could not be reconstructed reliably from the available retrospective extraction. These limitations constrain detailed time-to-event and procedure-pathway interpretation.

## 5. Conclusions

In this retrospective EOS cohort, MCGR treatment was associated with improved Cobb angle, measured radiographic T1–S1 length, and AVT through three years among patients with available measurements. The preoperative-to-year-3 T1–S1 difference included the effect of index surgery and should not be interpreted as biological spinal growth alone. Year-3 response estimates were conditional on imaging availability, and revision/fusion fields were not mutually exclusive. Given the incomplete follow-up and small number of unplanned revision events, these findings should be interpreted as descriptive and hypothesis-generating rather than definitive evidence of treatment efficacy or risk.

## Figures and Tables

**Table 1:** Baseline characteristics and radiological follow-up availability.

Variable		n	Summary
Total patients		55	
Sex	Female	33	33/55 (60.0%)
	Male	22	22/55 (40.0%)
Age at surgery (years)		55	8.10 ± 1.86; median 8.17 (Q1–Q3: 7.17–9.75); range 3.50–10.67
Etiology	Neuromuscular	23	23/55 (41.8%)
	Congenital	22	22/55 (40.0%)
	Idiopathic	7	7/55 (12.7%)
	Other/unclear	3	3/55 (5.5%)
Construct	Dual-rod	46	46/55 (83.6%)
	Single-rod	9	9/55 (16.4%)

Continuous variables are presented as mean ± standard deviation, with median (Q1–Q3) and range additionally reported for age. Categorical variables are presented as n (%). Age values entered in months were divided by 12 to obtain years.

**Table 2:** Serial radiographic summaries.

Outcome	Time Point	Available n	Mean ± SD	Median (Q1–Q3)	Missing n
T1–S1 length (mm)	Preoperative	55	325.60 ± 42.39	330.00 (298.00–352.00)	0
	First postoperative	55	340.84 ± 41.71	345.00 (313.00–362.00)	0
	Year 1	54	360.89 ± 47.68	371.00 (327.00–391.00)	1
	Year 2	54	363.43 ± 47.23	364.00 (330.25–397.50)	1
	Year 3	40	383.02 ± 54.42	380.50 (347.50–414.00)	15
AVT (mm)	Preoperative	55	53.36 ± 25.84	52.00 (36.00–67.50)	0
	First postoperative	55	44.56 ± 21.51	42.00 (29.50–59.00)	0
	Year 1	55	37.04 ± 20.20	33.00 (26.50–49.00)	0
	Year 2	54	34.59 ± 21.20	29.50 (21.00–46.50)	1
	Year 3	40	32.80 ± 23.56	28.00 (17.75–38.25)	15
Cobb angle (°)	Preoperative	55	63.85 ± 21.75	61.00 (46.00–81.00)	0
	First postoperative	55	55.29 ± 17.44	51.00 (41.50–68.50)	0
	Year 1	55	45.18 ± 16.36	43.00 (33.50–55.00)	0
	Year 2	54	41.30 ± 17.73	38.00 (29.25–54.00)	1
	Year 3	40	40.15 ± 19.03	35.00 (26.00–53.25)	15

Values are available-case summaries. SD: standard deviation; Q1–Q3: interquartile range; AVT: apical vertebral translation.

**Table 3:** GEE estimates of radiographic changes from preoperative measurements.

Outcome	Comparison	Estimate (95% CI)	Robust SE	*p*	Observations/Patients
Cobb angle (°)	Year 1 vs. preoperative	−18.67 (−22.51 to −14.84)	1.96	<0.001	204/55
	Year 2 vs. preoperative	−22.82 (−27.04 to −18.59)	2.15	<0.001	204/55
	Year 3 vs. preoperative	−23.70 (−29.36 to −18.04)	2.89	<0.001	204/55
T1–S1 length (mm)	Year 1 vs. preoperative	+35.36 (+26.90 to +43.82)	4.32	<0.001	203/55
	Year 2 vs. preoperative	+37.90 (+28.51 to +47.29)	4.79	<0.001	203/55
	Year 3 vs. preoperative	+53.06 (+41.43 to +64.70)	5.94	<0.001	203/55
AVT (mm)	Year 1 vs. preoperative	−16.33 (−20.74 to −11.92)	2.25	<0.001	204/55
	Year 2 vs. preoperative	−19.35 (−24.35 to −14.36)	2.55	<0.001	204/55
	Year 3 vs. preoperative	−21.11 (−27.38 to −14.85)	3.20	<0.001	204/55

Estimates were obtained using generalized estimating equations. Negative estimates indicate reductions in Cobb angle and AVT; positive estimates indicate increases in T1-S1 length. SE: standard error; CI: confidence interval.

**Table 4:** Year-3 radiographic outcomes and exploratory response endpoints.

Outcome	n	Mean ± SD or n/N (%)	Median (Q1–Q3)
Continuous year-3 outcomes			
Cobb correction (°)	40	22.93 ± 20.70	20.00 (9.75–35.25)
Cobb correction (%)	40	33.60 ± 28.33	37.27 (15.78–54.66)
Preoperative-to-year-3 T1–S1 length difference (mm)	40	53.75 ± 40.01	58.00 (19.00–84.25)
First-postoperative-to-year-3 T1–S1 length difference (mm)	40	36.53 ± 37.73	39.00 (6.75–58.00)
AVT improvement (mm)	40	20.33 ± 22.36	19.50 (4.25–31.25)
Exploratory responder endpoints			
Cobb correction ≥ 20°	40	20/40 (50.0%)	—
Cobb correction ≥ 30%	40	22/40 (55.0%)	—
Preoperative-to-year-3 T1–S1 length difference ≥ 30 mm	40	27/40 (67.5%)	—
Both ≥30% Cobb correction and ≥30 mm T1–S1 length difference	40	14/40 (35.0%)	—
Both radiographic thresholds and no recorded unplanned revision	40	10/40 (25.0%)	—

Year-3 outcomes were calculated among the 40 patients with numeric Cobb angle, T1-S1 length, and AVT measurements at year 3. T1–S1 differences are measured length differences; the preoperative-to-year-3 value includes index surgical correction and should not be interpreted as biological spinal growth alone. Responder cutoffs are exploratory. AVT: apical vertebral translation.

**Table 5:** Patient-level complications and revision records.

Section	Recorded Outcome	n	Summary
Complications	Any recorded complication	11	11/55 (20.0%)
	Infection-related	8	8/55 (14.5%); 8/11 complications
	Screw pull-out	2	2/55 (3.6%); 2/11 complications
	Rod fracture	1	1/55 (1.8%); 1/11 complications
Complication management	Debridement	6	6/11 (54.5%)
	Antibiotics	2	2/11 (18.2%)
	Screw revision	2	2/11 (18.2%)
	Rod exchange	1	1/11 (9.1%)
Unplanned revision	Unplanned revision recorded as “Yes”	9	9/55 (16.4%)
	Superficial infection/wound complication indication	6	6/9 unplanned revision indications
	Screw pull-out indication	2	2/9 unplanned revision indications
	Rod fracture indication	1	1/9 unplanned revision indications
Revision classification field	Internal revision recorded	24	24/55 (43.6%)
	External revision recorded	7	7/55 (12.7%)
	No internal/external revision recorded	24	24/55 (43.6%)
Procedure-type field	Rod exchange recorded	16	16/55 (29.1%)
	System exchange recorded	6	6/55 (10.9%)
Definitive fusion field	Definitive fusion recorded as “Yes”	9	9/55 (16.4%)

Values are patient-level counts from the 55 patient rows. Complication-management percentages use the 11 patients with recorded complications; indication percentages use the 9 patients flagged for unplanned revision. Revision classification, procedure type, unplanned revision, and definitive fusion are separate fields and may overlap. Procedure-type counts are reported as recorded and are not treated as verified counts of planned post-index procedures.

**Table 6:** Exploratory outcomes by scoliosis etiology.

Etiology	N	Year-3 Radiographs Available	Any Complication	Internal Revision Recorded	Unplanned Revision Recorded	Definitive Fusion Recorded
Neuromuscular	23	18/23 (78.3%)	4/23 (17.4%)	9/23 (39.1%)	3/23 (13.0%)	5/23 (21.7%)
Congenital	22	16/22 (72.7%)	4/22 (18.2%)	10/22 (45.5%)	3/22 (13.6%)	3/22 (13.6%)
Idiopathic	7	6/7 (85.7%)	3/7 (42.9%)	5/7 (71.4%)	3/7 (42.9%)	1/7 (14.3%)
Other/unclear	3	0/3 (0.0%)	0/3 (0.0%)	0/3 (0.0%)	0/3 (0.0%)	0/3 (0.0%)

Values are n/N (%). Revision and fusion indicators are separately recorded and may overlap. These subgroup summaries are exploratory; no inferential comparisons were performed because of the small group sizes.

**Table 7:** Characteristics and recorded outcomes by year-3 radiographic availability.

Variable	Year-3 Radiographs Available (n = 40)	Year-3 Radiographs Unavailable (n = 15)
Age at surgery, years	8.30 ± 1.86	7.56 ± 1.81
Sex, n (%)		
Female	25 (62.5%)	8 (53.3%)
Male	15 (37.5%)	7 (46.7%)
Etiology, n (%)		
Neuromuscular	18 (45.0%)	5 (33.3%)
Congenital	16 (40.0%)	6 (40.0%)
Idiopathic	6 (15.0%)	1 (6.7%)
Other/unclear	0/0.0%	3/15 (20.0%)
Rod configuration, n (%)		
Dual rod	34 (85.0%)	12 (80.0%)
Single rod	6 (15.0%)	3 (20.0%)
Preoperative radiographic measurements, mean ± SD		
Cobb angle (°)	63.08 ± 22.67	65.93 ± 19.69
T1–S1 length (mm)	329.28 ± 43.17	315.80 ± 40.01
AVT (mm)	53.13 ± 26.79	54.00 ± 23.99
Recorded follow-up duration, months	45.38 ± 13.11	27.07 ± 4.03
Recorded outcomes, n (%)		
Any complication	8 (20.0%)	3 (20.0%)
Internal revision classification	21 (52.5%)	3 (20.0%)
External revision classification	5 (12.5%)	2 (13.3%)
No revision classification recorded	14 (35.0%)	10 (66.7%)
Unplanned revision recorded	7 (17.5%)	2 (13.3%)
Definitive fusion recorded	9 (22.5%)	0

Values are mean ± SD or n (%), calculated within each column. Age was converted from the spreadsheet’s age field, recorded in months, to years. The age field showed inconsistencies with age calculated from the recorded dates, so this comparison should be interpreted cautiously. Revision categories and outcome flags are reported as recorded; no inferential comparisons were performed.

**Table 8:** Exploratory univariable Cox proportional hazards analyses of unplanned revision.

Predictor	Hazard Ratio (95% CI)	*p* Value
Preoperative Cobb angle ≥ 60° vs. <60°	8.30 (1.04–66.44)	0.046
Preoperative Cobb angle, per 10° increase	1.28 (0.96–1.69)	0.090

The predictors were evaluated in separate univariable Cox models. The primary analysis included nine events. CI: confidence interval. The analyses are exploratory and hypothesis-generating.

## Data Availability

The data supporting the findings of this study are available from the corresponding author upon reasonable request, subject to institutional and ethical restrictions.

## Supplementary Materials

The following supporting information can be downloaded at: https://www.mdpi.com/article/10.3390/jcm15208049/s1, Table S1: Paired changes from preoperative measurements.

## Abbreviations

AVT	Apical vertebral translation
CI	Confidence interval
EOS	Early-onset scoliosis
GEE	Generalized estimating equations
HR	Hazard ratio
MAGEC	Magnetic Expansion Control
MCGR	Magnetically controlled growing rod
OR	Odds ratio
Q1–Q3	First and third quartiles
SD	Standard deviation
SE	Standard error
T1–S1	First thoracic vertebra to first sacral vertebra length
