# Supplementary material for: Longitudinal Radiographic Outcomes and Revision Burden After Magnetically Controlled Growing Rod Treatment for Early-Onset Scoliosis
Source: J Clin Med. 2026 Oct 16;15(20):8049. doi: 10.3390/jcm15208049 (PMC13705815; doi:10.3390/jcm15208049)
Supplement: Supplementary file 1 [file jcm-15-08049-s001.zip › jcm-4518061-supplementary.pdf]

Supplemantary Table S1. Paired changes from preoperative measurements

Outcome	Comparison	Paired n	Median paired change (Q1-Q3)	Wilcoxon W	Holm-adjusted p
Cobb angle (°)	First postoperative vs preoperative	55	-9.00 (-12.00 to -1.00)	3.0	<0.001
	Year 1 vs preoperative	55	-19.00 (-26.50 to -7.50)	32.0	<0.001
	Year 2 vs preoperative	54	-23.50 (-33.75 to -11.50)	22.5	<0.001
	Year 3 vs preoperative	40	-20.00 (-35.25 to -9.75)	37.0	<0.001
T1-S1 length (mm)	First postoperative vs preoperative	55	14.00 (5.00 to 23.50)	29.5	<0.001
	Year 1 vs preoperative	54	38.50 (18.25 to 53.00)	86.0	<0.001
	Year 2 vs preoperative	54	29.00 (12.50 to 66.50)	80.0	<0.001
	Year 3 vs preoperative	40	58.00 (19.00 to 84.25)	14.5	<0.001
AVT (mm)	First postoperative vs preoperative	55	-8.00 (-13.50 to -0.50)	0.0	<0.001
	Year 1 vs preoperative	55	-16.00 (-26.00 to -4.00)	116.0	<0.001
	Year 2 vs preoperative	54	-18.50 (-32.75 to -4.25)	78.5	<0.001
	Year 3 vs preoperative	40	-19.50 (-31.25 to -4.25)	70.5	<0.001

Change was calculated as postoperative minus preoperative value. Negative values indicate reductions in Cobb angle or AVT; positive values indicate an increase in T1-S1 length. Wilcoxon signed-rank tests were used. P values were Holm-adjusted across the four postoperative comparisons within each outcome. Q1-Q3: interquartile range; AVT: apical vertebral translation.
